# Surgical treatment of fracture sequelae of the proximal humerus according to a pathology-based modification of the Boileau classification results in improved clinical outcome after shoulder arthroplasty

**DOI:** 10.1007/s00590-023-03721-9

**Published:** 2023-09-10

**Authors:** Michael Kimmeyer, Jonas Schmalzl, Evelin Schmidt, Annika Graf, Verena Rentschler, Christian Gerhardt, Lars-Johannes Lehmann

**Affiliations:** 1 Department of Traumatology, Hand Surgery and Sports Medicine, ViDia Clinics Karlsruhe, Steinhaeusserstr. 18, 76135 Karlsruhe, Germany; 2 Alps Surgery Institute, Clinique Générale Annecy, 4 Chemin de La Tour la Reine, 74000 Annecy, France; 3 Department of Trauma, Hand, Plastic and Reconstructive Surgery, University Hospital Wuerzburg, Oberduerrbacher Str. 6, 97080 Würzburg, Germany; 4 University of Jena, Bachstr. 18, 07743 Jena, Germany

**Keywords:** Avascular head necrosis, Locked shoulder dislocation, Pseudarthrosis, Anatomic shoulder arthroplasty, Total shoulder arthroplasty, Reverse shoulder arthroplasty, Bone graft augmentation, Pectorals major tendon transfer

## Abstract

**Background:**

Fracture sequelae of the proximal humerus were classified by Boileau into four types. Since there are pathomorphological differences and specific characteristics within the four types, we have developed a subclassification. For elderly patients, shoulder arthroplasty is mostly recommended. Based on the available literature and clinical trial results, a subclassification could be created that suggests a specific therapy for each subgroup. The aim of this study was to evaluate the endoprosthetic therapy according to the proposed subclassification and to provide an overview of the clinical and radiological results after endoprosthetic treatment of proximal humerus fracture sequelae.

**Methods:**

Patients with fracture sequelae of the proximal humerus who underwent arthroplasty according to the suggestion of the subclassification were included. Minimum time to follow-up was twelve months. General condition and several specific shoulder scores as the Constant–Murley Score (CS) were recorded at the follow-up examination. Complication and revision rates were analyzed.

**Results:**

In total, 59 patients (72.6 ± 10.0 years, 47 females, 12 males) were included. Mean follow-up time was 31.3 ± 17.0 months. Reverse shoulder arthroplasty was performed in 49 patients and anatomic shoulder arthroplasty was performed in ten patients. The CS increased by 47.3 points from preoperative (15.0) to postoperative (62.3). Good or very good clinical results were seen in 61% of the patients. Complications were observed in twelve (20%) patients and revision surgery was performed in nine (15%) patients.

**Conclusion:**

Due to of the variety of fracture sequelae of the proximal humerus, a modification of the Boileau classification seems necessary. This study shows that endoprosthetic treatment for fracture sequelae can significantly improve the shoulder function in elderly patients. Good clinical results can be achieved with a comparatively low revision rate following the treatment suggestions of the proposed subclassification of the Boileau classification.

**Level of evidence IV:**

Case series.

## Background

Proximal humerus fractures account for 4–6% of all fractures [8]. They occur more often in older people and are more common in women [19]. A simple fall can be a sufficient cause in patients with poorer bone quality. Due to demographic transition, a further increase in proximal humerus fractures is expected in the coming years [20]. Conservative therapy can be recommended for the majority of proximal humerus fractures, while dislocated fractures are often treated surgically [7]. The specific treatment decision is based on fracture morphology, bone-quality and patient-specific criteria as age, physical activity and comorbidities. The optimal treatment option is still controversial. Both conservatively and surgically treated proximal humerus fractures have a very high complication rate [5, 6, 27]. The indication for the appropriate therapy and the technical aspect of the surgery is very demanding and high rates of complications of up to 45% were reported [14, 16, 22].

**Fig. 1:**
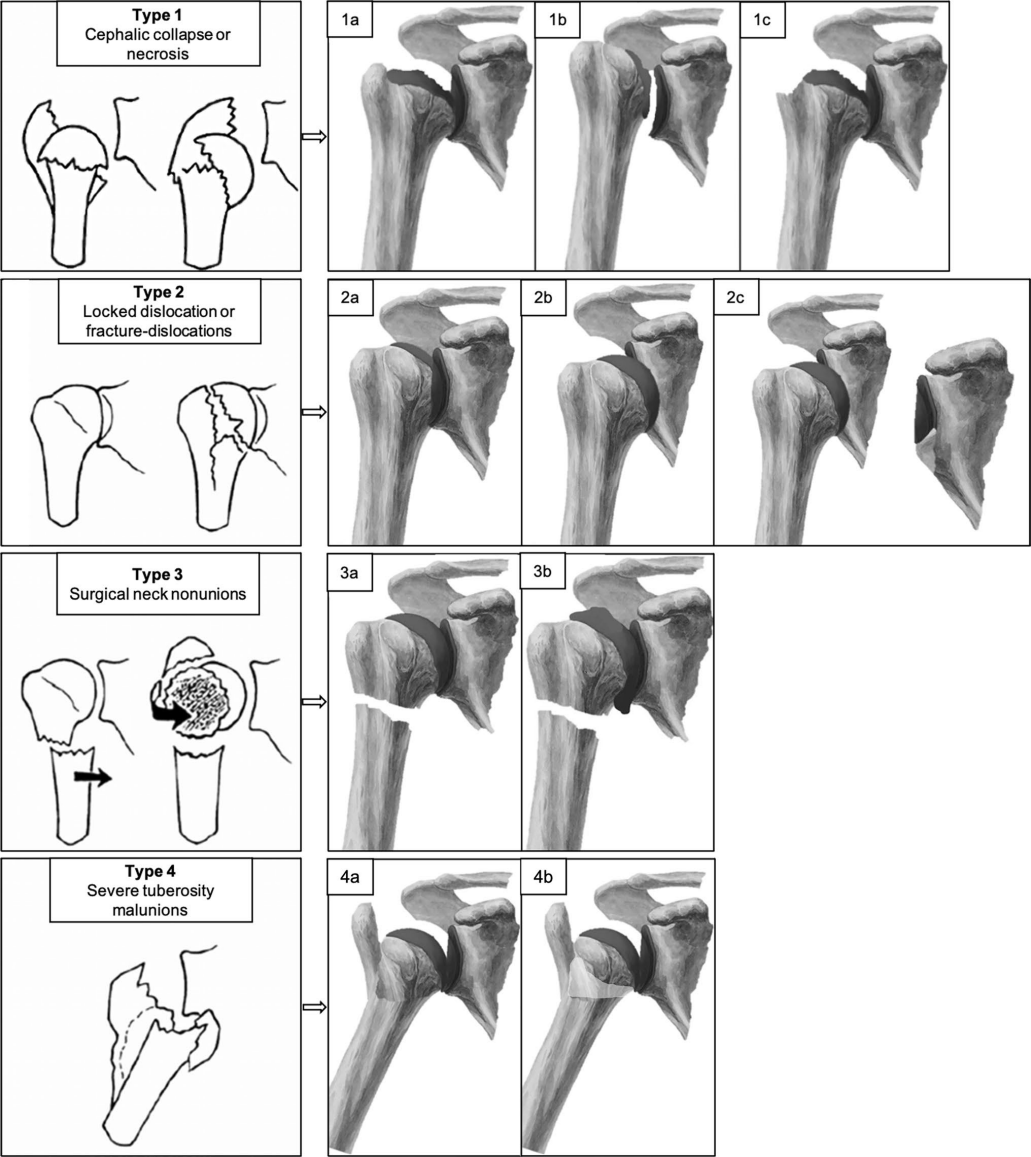
Fracture sequela of the proximal humerus and modification of the Boileau classification types 1a-c, types 2a-c, types 3a-b and type 4-b. (Image source: Schmalzl et al., BMC Musculoskeletal Disorders, 2022, https://doi.org/10.1186/s12891-022-05338-1; Schmalzl et al., European Journal of Orthopaedic Surgery & Traumatology, 2021, https://doi.org/10.1007/s00590-021-03022-z)

Boileau et al. analysed fracture sequelae of the proximal humerus and divided them into four types, distinguishing between intra- (type 1 and 2) and extraarticular (type 3 and 4) lesions [4]. Type 1 are fracture sequelae with avascular necrosis or collapse of the humeral head (Fig. 1). Type 2 are chronic locked dislocations or chronic locked fracture dislocations (Fig. 1). Type 3 are pseudarthroses of the surgical neck (Fig. 1). Type 4 are severe deformities of the greater tuberosity (Fig. 1). There are various surgical strategies to treat the different types of fracture sequelae. Basically, a distinction must be made between joint-preserving and joint-replacing procedures. In elderly patients, endoprosthetic surgery is mostly performed. The indication for the appropriate therapy and the technical aspect of the surgery is very demanding and high rates of complications of up to 45% were reported [14, 16, 22]. The classification by Boileau et al. gives a good overview of the most common fracture sequelae after proximal humerus fracture and shows the results after performing the ASA. In the original publications, the RSA was not included in the analysis. However, since the RSA has shown good clinical results in the treatment of fracture sequelae in other studies, it should definitely be considered. In addition, after analyzing our patients, we found several morphological differences within the types and proposed a subclassification [22–24]. Comparing our postoperative results according to the subclassification and analyzing the available literature, we were able to identify an advantageous surgical procedure for each type. The aim of this study to provide an overview of the postoperative results after the suggested endoprosthetic treatment of proximal humerus fracture sequelae according.

## Material and methods

This study is a retrospective case series. The patients were operated in a certified center of shoulder and elbow surgery. The surgeries took place between July 2014 and September 2020. The data were collected after a signed declaration of consent. A positive vote from the responsible ethics committee is available. The follow-up examination respect the ethical standards in the Helsinki Declaration of 1975, as revised in 2000, as well as the national law.

### Study population

**Fig. 2:**
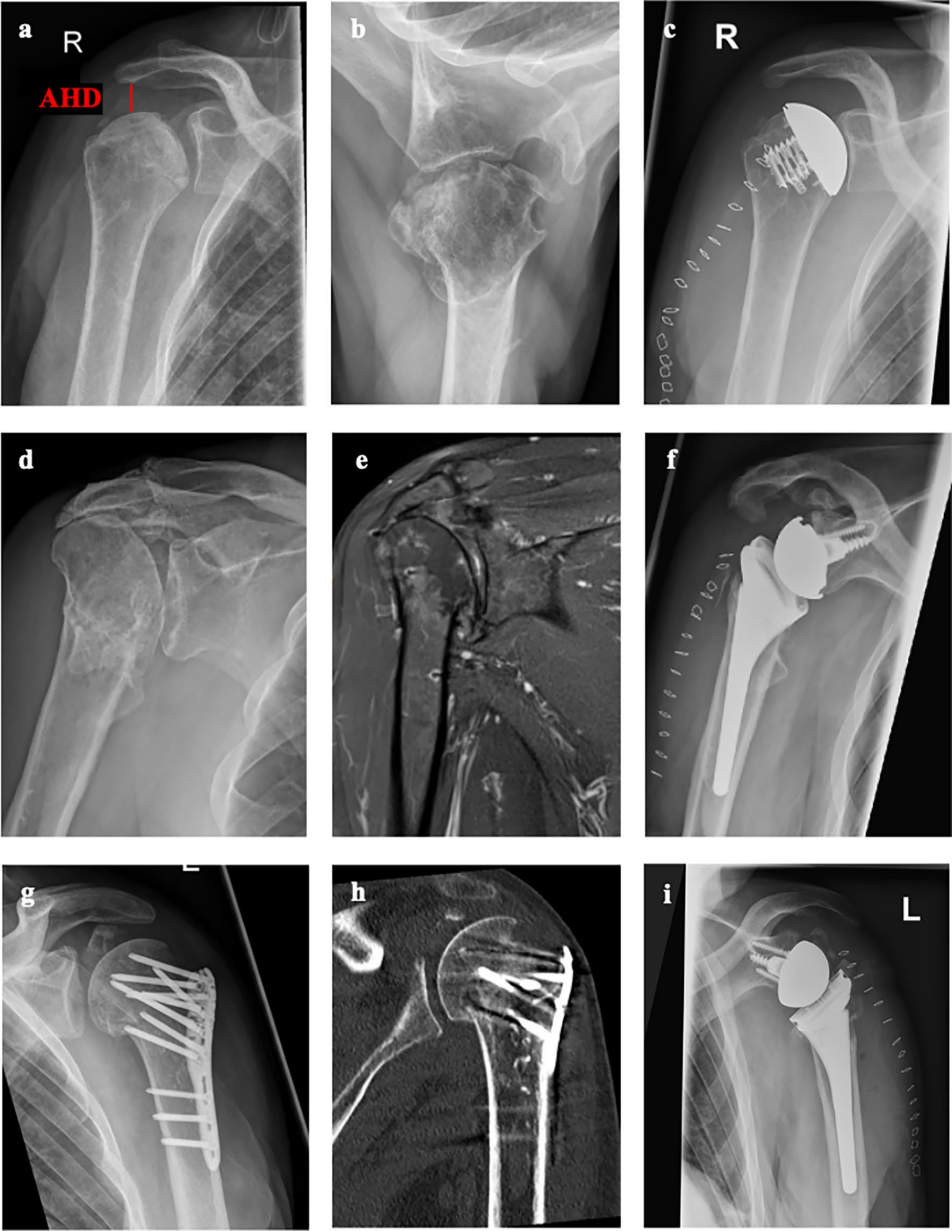
Overview of the subclassification of fracture sequelae type 1 of the proximal humerus based on radiological imaging and presentation of the corresponding therapy recommendations: **a**–**c**) type 1a with AHD > 7 mm treated with AHSA; **d**–**f** type 1b treated with RSA; **g**–**i**) type 1c treated with RSA. (AHD = acromiohumeral distance, AHSA = Anatomical Hemi Shoulder Arthroplasty, RSA = Reverse Shoulder Arthroplasty)

**Fig. 3:**
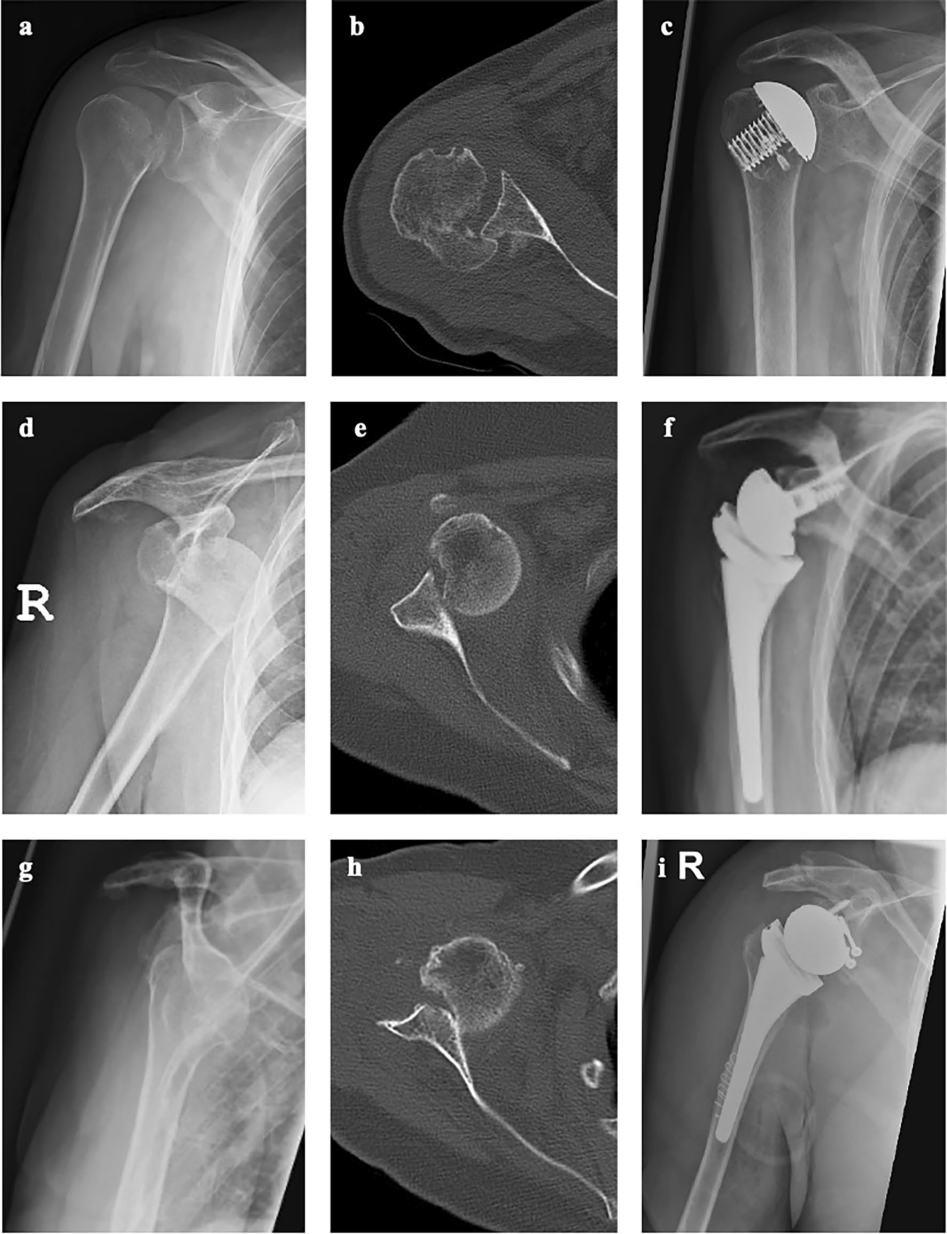
Overview of the subclassification of fracture sequelae type 2 of the proximal humerus based on radiological imaging and presentation of the corresponding therapy recommendations: **a**–**c** type 2a treated with AHSA and refixation of the subscapularis tendon with suture anchor; **d**–**f** type 2b treated with RSA; **g**–**i** type 2c treated with RSA and bony glenoid augmentation and fixation with two screws. (AHSA = Anatomical Hemi Shoulder Arthroplasty, RSA = Reverse Shoulder Arthroplasty)

Included were all patients with fracture sequelae of the proximal humerus according to Boileau classification surgery according to the suggestions of our subclassification (Figs. 2, 3). Patients treated with a surgical procedure different from that proposed in the subclassification were excluded accordingly. The follow-up period was at least twelve months, and the minimum age was 50 years. All patients were required to present for clinical and radiographic examination. Patients who could not attend follow-up for medical reasons, who could not be contacted, or who did not sign an informed consent form were excluded.

### Subclassification and treatment suggestion

Type 1 lesions are subclassified into three groups (Figs. 1, 2). In type 1a lesions with an adequate acromiohumeral distance (AHD) of more than 7 mm, an anatomic total shoulder arthroplasty (ATSA) or anatomic hemi shoulder arthroplasty (AHSA) is recommended. In type 1b lesions with a reduced AHD of less than 7 mm and in type 1c lesions with a posttraumatic osteonecrosis of the humeral head and resorption of the greater tuberosity, a reverse shoulder arthroplasty (RSA) should be used because of rotator cuff dysfunction. Type 2 lesions were also divided into three groups (Figs. 1, 3). Chronic posterior locked dislocations (type 2a) can be treated with ATSA or AHSA. Chronic anterior locked dislocations (type 2b) should be treated with RSA, and in case of a bony glenoid defect (type 2c) of more than 30%, autologous bone grafting with the resected humeral head should be performed. For type 3 lesions, a differentiation between cases without preexisting glenohumeral osteoarthritis or cuff tear arthropathy (type 3a) and cases with corresponding degenerative changes (type 3b) were made. For type 3a lesion head-preserving therapy approaches should be preferred. In type 3b lesions RSA might be the best option. In type 4 lesions, RSA seems to be a reasonable treatment option. Depending on a metaphyseal bone defect, bone grafting should be considered (type 4b). In younger patients, anatomic joint replacement may also be considered, but successful healing of the osteotomized greater tuberosity is critical.

### Surgical treatment

The surgeries were performed under general and/or regional anesthesia using an interscalene plexus block in beach-chair position. A deltopectoral approach was used in every case. For anatomic shoulder arthroplasty (ASA), a stemless implant was used (Eclipse®, Arthrex®, Naples, FL, USA). Depending on the cartilage status of the glenoid, the glenoid was replaced. The subscapularis tendon was reattached with FiberWire® sutures (Arthrex®). In this study, two different implants were used as RSA. In fracture sequelae of the proximal humerus with bony glenoid defects below 40% of the articular surface, a cementless implant with a humeral inclination of 135° and a lateralization of 4 mm was chosen (Univers Revers®, Arthrex®). In the case of bony glenoid defects larger than 40% of the articular surface, an RSA with a central screw of 30 mm was used (Altivate Reverse Shoulder Prosthesis®, DJO®, Dallas, TX, USA). The humeral inclination of this prosthesis was also 135°. Depending on the extent and localization of the bony glenoid defect, an autologous bone augmentation was performed using the resected humeral head. Fixation of the bone graft was either achieved via the screws of the baseplate or separate cannulated screws (CCS SpeedTip®, 3 mm, Medartis®, Basel, Switzerland). Depending on the condition of the subscapularis tendon, the tendon was reattached with FiberWire® sutures (Arthrex®). Optionally, after implantation of the humeral component, the tendon of the pectoralis major muscle was detached from its attachment to the crista tuberculi majoris humeri and refixed transosseously to the lesser tuberosity. Figures 2 and 3 show an overview of cases with fracture sequelae type 1 and 2 including the corresponding surgical therapy recommendation.

The postoperative treatment included an early functional therapy or immobilization in a shoulder abduction splint for three weeks. The restrictive protocol was used in case of a refixation of the subscapularis tendon or tendon transfer. In these cases, the patients were allowed to start active movements after six weeks.

### Functional outcomes

At follow-up, active and passive range of motion (ROM) of the shoulder (abduction, flexion, external rotation, internal rotation) were evaluated. The isometric abduction strength was measured in 90° in the scapular plane. Two measurements were made per side and the mean value was calculated. Several specific shoulder scores were evaluated: Constant-Murley Score (CS), age and gender adjusted CS (ACS), Subjective Shoulder Value (SSV), Quick Disabilities of the Arm, Shoulder and Hand Score (QDASH) and pain on the Visual Analog Scale (VAS). General condition at follow-up was measured by European Quality Visual Analog Scale (EQ-VAS). The preoperative CS was determined from the data collection of the endoprosthesis register.

### Radiological outcomes, complications and revision surgery

Preoperative computed tomography (CT) scans as well as pre- and postoperative radiographs (true anterior posterior (ap) view, lateral (Y) view) were evaluated. In preoperative CT scans the fracture sequelae (Boileau and modified Boileau classification), the grade of glenohumeral osteoarthritis (Samilson-Prieto [21]), the morphology of the glenoid (Walch [28]), the extent of the bony glenoid defect, the direction of the dislocation and the integrity of the greater tuberosity were analyzed. In ap radiographs, the AHD was measured (Fig. 2). Based on radiographs at follow-up, prosthesis position, scapular notching according to Sirveaux [26], reduction or dislocation, or resorption of the greater tuberosity were analyzed. A secondary osteoarthritis in patients with AHSA was defined as a complication if the osteoarthritis of the glenoid was progressive and painful. Further complications such as hematoma, current and chronic infection, prosthesis loosening, implant dissociation, dislocation, periprosthetic fracture, nerve injury, and revision surgery were also recorded.

### Statistical evaluation

The patients were divided into groups according to the modified Boileau classification. The ACS was ranked in groups according to the clinical outcome: “very good” (86–100), “good” (71–85), “fair” (56–70), “poor” (< 56) [2]. Statistical analyses were performed using SPSS ® software (version 28.0; IBM ®, Armonk, United States of America). The nominal variables were summarized as percentages. The arithmetic mean and its standard deviation were used for descriptive statistics. Continuous and ordinal variables were grouped with medians and ranges. Shapiro–Wilk test was used to test the normality of the variables. Wilcoxon-Mann–Whitney test (U test) was used for quantitative variables based on distribution normality. For testing the association of two ordinal variables, the Pearson-χ [2]-test was calculated. The Odds’ ratio was performed to quantify the association of qualitative variables. For exploratory statistics, the level of significance was set for *p* < 0.05.

## Results

A total of 103 patients with fracture sequelae of the proximal humerus were treated with arthroplasty during the defined period (14 type 1a, 26 type 1b, 14 type 1c, 7 type 2a, 15 type 2b, 11 type 2c, 2 type 3b, 14 type 4a). Eight patients were not operated on according to the subclassification suggestion and were therefore not included in the analysis. Patients could not be reexamined due to unavailability (1), disability or illness (3) or death (17). A total of 15 patients did not want to participate in the study. Thus, loss to follow-up was 38%.

In total, 59 patients (female = 47 (80%), male = 12 (20%) were included. 32 (54%) type 1 lesions, 17 (29%) type 2 lesions, one (3%) type 3 lesion, and nine (15%) type 4 lesions were identified. RSA was performed in 49 patients and ASA in ten patients (6 AHSA, 4 ATSA). Mean follow-up time was 31.3 ± 17.0 months and mean age at follow-up was 72.6 ± 10.0 years. The EQ-VAS was 67.5 ± 20.6%. The general parameters of the study population are shown in Table 1.

**Table 1:** Study population according to the type of prosthesis

Study population	RSA	ASA
RSA (*n* = 49)		
RSA 1	43 (87.8%)	
RSA 2	6 (12.2%)	
ASA (*n* = 10)		
AHSA		6 (60.0%)
ATSA		4 (40.0%)
Refixation SSC		
Yes	16 (32.7%)	10 (100.0%)
No	33 (67.3%)	0 (0.0%)
PMT		
Yes	9 (18.4%)	0 (0.0%)
No	40 (81.6%)	10 (100.0%)
Glenoid bone graft		
Yes	7 (14.3%)	0 (0.0%)
No	32 (85.7%)	10 (100.0%)
Age		
Years	74.5 ± 9.2	63.4 ± 8.9
Follow-up time		
Months	29.6 ± 16.5	39.5 ± 18.3
Gender		
Female	40 (81.6%)	7 (70.0%)
Male	9 (18.4%)	3 (30.0%)
BMI		
kg/cm^2^	28.1 ± 4.9	28.1 ± 6.0
Smoking		
Yes	5 (10.2%)	6 (60.0%)
No	44 (89.8%)	4 (40.0%)
Diabetes mellitus		
Yes	13 (26.5%)	0 (0.0%)
No	36 (73.5%)	11 (100.0%)

RSA = reverse shoulder arthroplasty, ASA = anatomic shoulder arthroplasty, RSA 1 = Univers Revers® (Arthrex®, Naples, FL, USA), RSA 2 = Altivate Reverse Shoulder Prosthesis® (DJO®, Dallas, TX, USA), AHSA = anatomic hemi shoulder arthroplasty, ATSA = anatomy total shoulder arthroplasty, SSC = subscapularis tendon, PMT = pectoralis major tendon transfer, BMI = body mass index, kg = kilogramm, cm = centimeter

**Fig. 4:**
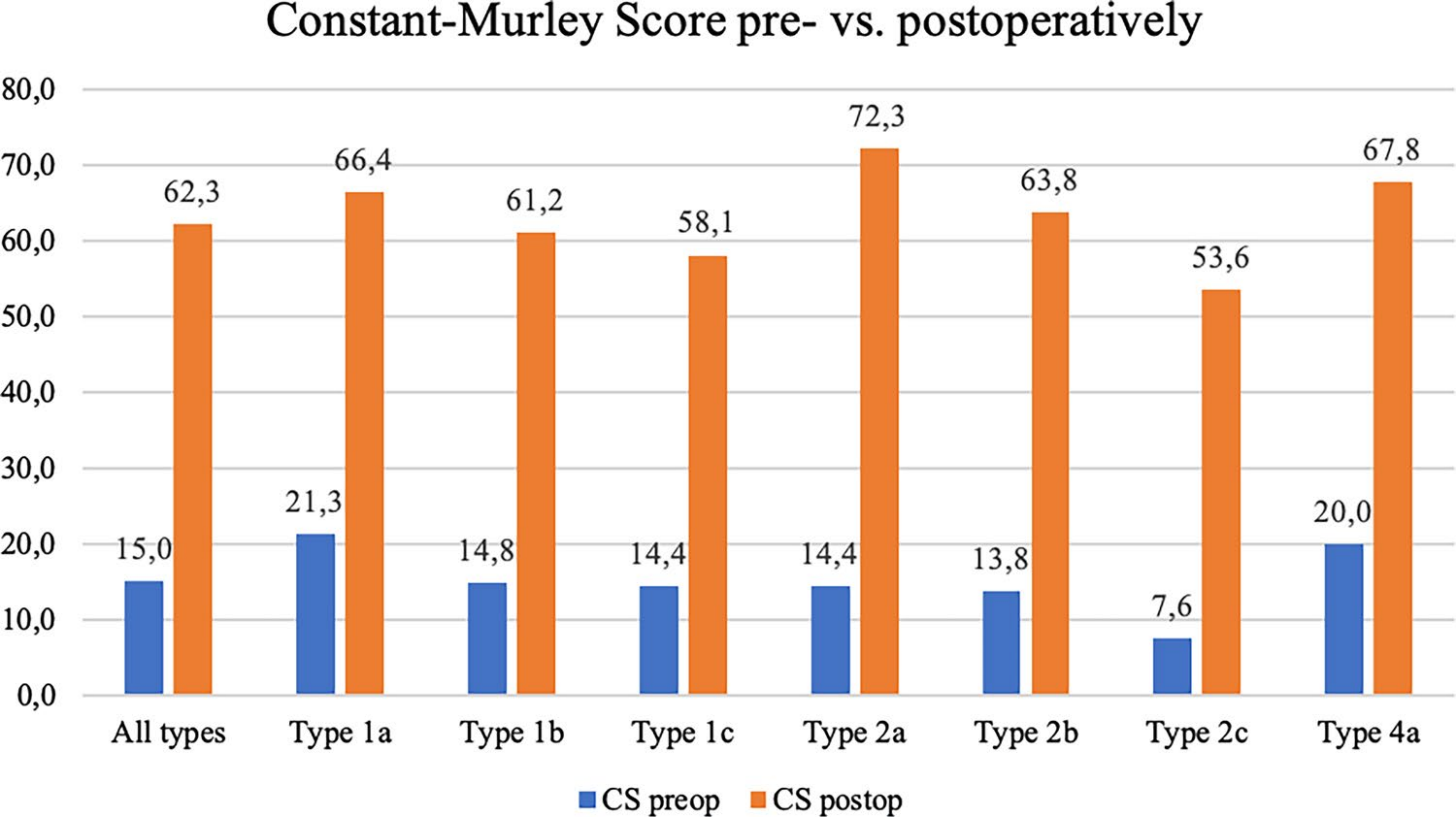
Evolution of the Constant-Murley Score pre- to postoperatively. (CS = Constant-Murley Score)

The functional outcome of the study population is presented in Table 2. Following the therapy suggestion of our subclassification, the CS increased by 47.3 points from preoperative to postoperative (Fig. 4). According to ACS, 36 (61%) patients had a good or very good functional outcome. An active internal rotation of at least the fifth lumbar vertebra was achieved by 53 (88%) patients. Complications were seen in twelve (20%) patients and revision surgery was performed in nine (15%) patients. Advanced scapular notching was not observed in any RSA. Patients with complications had a higher risk of a poorer functional outcome (Odds’ ratio 4.8) and patients without revision surgery had significantly better functional outcomes (CS 65.3 vs. 45.7, *p* = 0.002). Complication and revision rates in patients with a bone graft were 14% and 0%, respectively. Patients with PMT showed higher complication (33% vs. 15%, *p* = 0.477) and revision rates (22% vs. 12%, *p* = 0.528) rates without statistical significance.

**Table 2:** Clinical and functional outcome parameters according to the subclassification of fracture sequelae of the proximal humerus

Functional outcome		All	Type 1a	Type 1b	Type 1c	Type 2a	Type 2b	Type 2c	Type 3b	Type 4a
n		59	7	17	8	3	5	9	1	9
SSV	%	69.3 ± 18.0	65.9 ± 12.5	69.7 ± 16.2	65.0 ± 18.5	80.0 ± 13.2	78.0 ± 8.4	58.3 ± 28.1	75.0	76.9 ± 14.7
VAS		1.9 ± 2.2	1.7 ± 2.2	2.2 ± 1.8	1.9 ± 2.6	0.0 ± 0.0	2.0 ± 2.7	2.3 ± 2.6	0.0	1.7 ± 2.7
CS		62.3 ± 17.8	66.4 ± 18.5	61.2 ± 18.0	58.1 ± 14.2	72.3 ± 9.3	63.8 ± 20.6	53.6 ± 22.4	79.0	67.8 ± 15.7
ACS	%	68.2 ± 16.6	73.2 ± 13.4	65.8 ± 16.9	70.1 ± 13.6	78.9 ± 10.7	69.6 ± 19.2	59.7 ± 22.3	84.5	69.2 ± 14.9
CS preop		15.0 ± 8.8	21.3 ± 12.5	14.8 ± 7.3	14.4 ± 7.4	14.3 ± 5.5	13.8 ± 10.4	7.6 ± 1.3	8.0	20.0 ± 9.5
QDASH		20.6 ± 19.0	17.2 ± 14.2	21.9 ± 18.8	21.0 ± 18.9	4.5 ± 4.5	29.3 ± 27.0	27.3 ± 21.1	4.5	16.2 ± 18.8
Abduction		111.1 ± 35.1	115.0 ± 38.6	11.5 ± 35.8	113.8 ± 27.9	126.7 ± 32.1	110.0 ± 38.1	94.4 ± 41.9	150.0	112.8 ± 35.1
Flexion		120.3 ± 32.3	128.6 ± 26.7	118.8 ± 43.4	122.5 ± 26.6	133.3 ± 37.9	124.0 ± 23.0	106.7 ± 36.3	130.0	120.6 ± 41.6
ER		36.0 ± 19.1	50.0 ± 11.5	37.9 ± 23.1	27.5 ± 16.7	53.3 ± 11.5	20.0 ± 14.1	36.7 ± 12.2	60.0	28.9 ± 17.6

n = number, SSV = Subjective Shoulder Value, VAS = Visual Analog Scale, CS = Constant-Murley Score, ACS = age and sex corrected Constant-Murley Score, CS preop = preoperativ Constant-Murley Score, QDASH = Quick Disability Arm, Shoulder and Hand Score, ER = external rotation

### Type 1 lesions (avascular necrosis, collapse of the humeral head)

All type 1a lesions were operated on with ASA (5 AHSA, 2 ATSA). Two (29%) cases required revision surgery. In one case of AHSA, revision surgery and conversion to RSA was performed after 23 months because of rotator cuff insufficiency. In the second case, a two-stage prosthesis change with conversion to an RSA was performed because of periprosthetic infection of the AHSA. All type 1b and type 1c lesions were operated on by RSA. The complication rate was 24% and the revision rate was 16% (1 acute periprosthetic infection, 1 periprosthetic fracture, 2 low-grade infections). Two patients experienced insufficiency fractures of the acromion and coracoid process, respectively, which were treated conservatively.

### Type 2 lesions (chronic dislocations, chronic dislocation fractures)

ASA was performed in type 2a lesions (2 ATSA, 1 AHSA) and RSA in type 2b and 2c lesions (10 Univers Revers®, 4 Altivate Reverse Shoulder Prosthesis®). Type 2a lesions showed an ACS of 79% and an increase of 58 points of the CS. In type 2b and 2c lesions, analysis has shown that a bony glenoid defect of > 40% was correlated with a reduced functional outcome, although no statistical significance was found. Complications were noted in three (18%) patients (1 acute periprosthetic infection, 1 low-grade infection, 1 glenosphere malposition), and revision surgery was performed in two (12%) cases (2 two-stage revision surgeries). The patient with the malposition of the glenosphere did not want a revision surgery. An autologous glenoid bone transplant was not associated with an increased risk of complications or revision surgery. Cases with pectoralis major tendon transfer had an increased complication rate (43% vs. 0%, *p* = 0.023) and revision rate (29% vs. 0%, *p* = 0.072) as well as significantly worse functional outcomes (ACS: 51.6 vs. 76.3, *p* = 0.014, SSV: 54.3 vs. 70.5, *p* = 0.043). In both revision cases, several previous operations and a peri-implant infection were already present before the fracture sequelae were treated with arthroplasty.

### Type 3 lesions (pseudarthrosis of the surgical neck)

Only one patient was identified in the type 3b lesion group, who underwent RSA surgery. The functional result is presented in Table 2. No complications and revision were noted.

### Type 4 lesions (severe deformities of the tuberosities)

For type 4a lesions, RSA was performed in all nine patients. Functional outcome is shown in Table 2. Complication and revision rate were 11%. Due to one periprosthetic fracture, one glenosphere dissociation and one inlay dissociation, a total of three revision procedures were performed in the revision case.

## Discussion

This study presents the postoperative results of a pathology that is rare in occurrence and at the same time technically very challenging. Despite excellent surgical technique, an excellent functional postoperative result is difficult to achieve. In the literature, only small groups are often described, so that no clear statement can be made about the best choice of surgical technique. Based on our analysis and previous studies, we were able to identify different pathomorphological features for the fracture sequelae and, depending on them, determine certain operative techniques with better postoperative results. In this retrospective case series, we included 59 patients with fracture sequelae of the proximal humerus who were treated with an arthroplasty according to our proposed subclassification. Overall, good clinical results could be achieved. ACS ranged from 66 to 69%. ACS was excellent in 7% of patients, good in 54%, fair in 14%, and poor in 25%.

In the publication by Boileau et al. in 2001, 71 patients with fracture sequelae of the proximal humerus were treated with ASA [4]. In this multicenter study, 40 (56%) type 1, nine (13%) type 2, six (8%) type 3, and 16 (23%) type 4 lesions were included. Functional outcome (ACS) was excellent in 16% of cases, good in 26%, fair in 25%, and poor in 33%. In 2006, Boileau et al. published a multicenter study with a total of 201 patients who underwent ASA for fracture sequelae of the proximal humerus [3]. Overall, good clinical-functional outcomes were shown in the short period after arthroplasty, with an average CS of 61 points, a complication rate of 32%, and a revision rate of 24%. Compared to the two studies by Boileau et al., our study collective shows similar and improved postoperative results when our therapy suggestion are followed. The distribution in our study was comparable, although we observed a 2.2-fold proportion of type 2 lesions in our study (28% vs. 13%). In addition, our cohort showed a lower rate of postoperative complications (20% vs. 27%).

Since fracture sequelae differ in their entity and localization (intra- and extracapsular), they should be discussed according to the type of lesion.

### Type 1

According to Boileau, type 1 lesions are intracapsular fractures with avascular necrosis of the humeral head [4]. Various treatment options are described in the literature. These include head-preserving operations such as tuberosity osteotomy as well as head-replacing therapy options such as ASA or RSA. ASA showed promising mid-term results for type 1 lesions, although some unsatisfactory results and a high complication rate have also been reported [3, 4]. Feeley et al. published a study on 64 cases with humeral head necrosis treated with AHSA or ATSA [9]. They have not seen any differences in outcome or ROM between AHSA and ATSA. The complication rate was significantly higher with ATSA (22%) than with AHSA (8%). According to their results, AHSA should be chosen if the condition of the glenoid cartilage allows it. Moineau et al. published a modified classification of posttraumatic humeral head necrosis treated by ASA [14]. A total of 55 patients were included in the study. They differentiated the type 1 lesions into four subgroups in order to be able to give better treatment recommendations. They concluded that a varus dislocation, a fatty degeneration of the rotator cuff and defects of the tubercula were associated with significantly poorer postoperative results. In these cases, they recommended the use of an RSA.

The first study of type 1 lesions treated with RSA was published by Raiss et al. in 2018 [15]. They included 38 patients with type 1 lesions associated with rotator cuff deficiency or shoulder stiffness. There was an increase in mean CS from 25 to 57 points and high patient satisfaction. They concluded that RSA is an effective treatment for patients with type 1 sequelae of proximal humerus fracture associated with rotator cuff weakness or shoulder stiffness.

Based on these data, we decided to divide type 1 lesions into three groups. Type 1a lesions have an AHD of more than 7 mm and intact tuberosities, so that an intact rotator cuff can be assumed. In a study by our research group, we were able to show that ASA is superior to RSA in type 1a lesions. In the present study, an ACS of 73% was achieved in type 1a lesions after ASA. CS increased by 45 points from preoperative to postoperative. Type 1b and 1c lesions have reduced AHD (< 7 mm) or tuberosity resorption indication an insufficient rotator cuff, so we recommend treatment with RSA in this population. In these groups, CS was also improved after RSA (type 1b: + 46 points and type 1c: + 44 points). Compared to the available literature, no increased complication (24%) or revision (16%) rates were observed in this group.

### Type 2

Type 2 lesions are chronic locked dislocations or locked dislocation fractures of the humeral head. For type 2 lesions, we believe that the direction of dislocation and the extent of the glenoid defect should be considered. By making this distinction, we were able to identify treatment suggestions.

Several studies have evaluated endoprosthetic therapy for type 2 lesions [13, 17, 30]. The studies have in common that the functional outcome can be improved with both ASA and RSA. However, high complication rates of 32% to 45% were observed in these studies.

In type 2a lesions, posterior locked shoulder dislocation is present. Studies by our group showed that glenoid defects are very rarely observed in dorsal dislocation, and treatment with an ASA can achieve better functional results as long as the rotator cuff is intact [11, 22].

Treatment with RSA is recommended for anterior locked dislocations without (2a) or with a bony glenoid defect (2c). Raiss et al. published a case series of 22 patients with chronic locked shoulder dislocation operated by RSA [17]. The CS increased from 14 to 47 points in this study. The complication rate was 32% and revision rate was 27%. In case of bony glenoid reconstruction, a glenoid component failure rate of 80% was observed. One reason for the high failure rate could have been the fixation of the graft, as only a central peg of 15 mm was used. However, the authors concluded that RSA could be a viable treatment option for chronic locked shoulder dislocations with concomitant rotator cuff lesions and an intact glenoid. In another study, Werner et al. published 21 cases with chronic locked anterior shoulder dislocation with concomitant bony glenoid defect. All patients underwent surgery using RSA and a bony glenoid transplant. Glenosphere dislocation was observed in two patients, which in one case was due to trauma. In the other case, the authors suspected inadequate fixation of the baseplate. They concluded that the central peg should be fixed at least 10 mm into the native bone and the baseplate should be placed on at least 50% of the native bone. In the surgical treatment of type 2c lesions with a bony glenoid defect of more than 40%, we made sure to use an RSA with a long central screw of 30 mm to achieve good fixation of the baseplate and bone graft. In most (89%) cases, a good position of the glenosphere was seen in the radiographic follow-up images. Nevertheless, in one case there was a glenosphere in malposition.

Compared with the results from the literature, we obtained comparable and tendentially better functional results in our case series. Recurrent dislocation was not observed at all in our case series. From our point of view, careful soft tissue management plays an important role. In case of chronic degeneration of the subscapularis tendon and anterior locked shoulder dislocation, PMT should be considered to ensure good recentering of the prosthesis. However, the patients with PMT in type 2c lesions showed a worse clinical outcome and an increased complication and revision rate. Several preoperative factors had an impact on the clinical outcome in these cases. In patients with type 2c lesions treated with a PMT, larger glenoid defects (56% vs. 35%) and, as described before, in the two revision cases, multiple previous surgeries and an infection before the endoprosthetic treatment were noted. Further studies are needed to demonstrate the benefit of PMT in the treatment of chronic shoulder dislocation.

### Type 3

With this type, it should first be emphasized that the results of this study cannot be used to make any statement about a therapy recommendation (1 patient). The theoretical suggestion was based on an analysis of the available literature. Various surgical treatment options as open reduction and internal fixation of the proximal part of the humerus using an intramedullary bone graft in combination with plate or nail or endoprosthetic treatment options have been described for the treatment of pseudarthrosis at the surgical neck [1, 10, 29]. However, clinical outcomes appear to be variable and inconsistent among joint-preserving treatment strategies. In the multicenter study by Boileau et al. published in 2006, type 3 lesions were treated with ASA [3]. The authors reported unsatisfactory results and a high complication rate (32%). They described this type of fracture sequelae as a relative contraindication for ASA. The largest case series with a total of 32 patients with type 3 lesions treated with RSA was published by Raiss et al. [16]. The mean CS increased from 14 to 47 points at a mean follow-up of four years. There were 13 complications (41%) leading to nine revision procedures (28%). The most common complication was dislocation of the RSA, which occurred in eleven of the patients (34%). A case series by Martinez et al. showed promising clinical results and a complication rate of 22% (2 wound infections and 2 dislocations) in patients treated with RSA [12]. Depending on the patient's age and physical demands and activity, joint-conserving therapeutic approaches seem to be an option for type 3 lesions in patients without glenohumeral osteoarthritis (type 3a). RSA is suggested for elderly people and those with pre-existing glenohumeral osteoarthritis or defect arthropathy (type 3b). A metaphyseal fracture pattern is more frequently observed in proximal humerus fractures with existing osteoarthritis, which could have biomechanical causes [25]. As this could be a biomechanical weakness and an increased risk of pseudarthrosis when treated with open reduction and internal fixation, we suggest to treat type 3b lesions with RSA. Regarding the surgical technique, the RSA should be implanted by threading the pseudarthrosis after resection of the joint surface at the anatomical neck. A resection of the complete pseudarthrosis including the head should be avoided as this leaves the surgeon without orientation for the implantation of the prosthesis and might result in inadequate postoperative results.

### Type 4

In the multicenter studies by Boileau et al. type 4 lesions were treated with ASA [3, 4]. Implantation of an ASA in chronically dissociated tuberosities is challenging. As a functioning rotator cuff is essential, a tuberosity osteotomy is usually required to ensure good shoulder function. In the series of 201 fracture sequelae of Boileau et al. [3], 19 type 4 lesions were present. In this group, a complication rate of 32% and a revision rate of 16% was seen. Type 4 lesions showed worse clinical outcomes compared to the other groups. Healing of the tuberosities was seen as a major problem. The authors concluded that RSA should be preferred for type 4 lesions. The only study currently available in the literature that examined only patients with type 4 fracture sequelae treated with RSA was published in 2016 by Raiss et al. [18]. In 42 analyzed cases with a mean follow-up of four years, the mean CS increased from 20 points preoperatively to 55 points postoperatively. The complication rate was only 9.5%. In our study, we were also able to show that the clinical outcomes with RSA in type 4 lesions are fair to good. The CS increased by 48 points and the complication rate was 11%. Therefore, our results confirm the conclusion of Raiss et al. that type 4a lesions can achieve good clinical results using RSA. Based on these results, we suggest the use of RSA in elderly patients for type 4a lesions. In our opinion, bone transplantation should be considered in patients with an additional metaphyseal bone defect. However, this was not necessary in our collective in any of the patients with a type 4 lesion.

## Limitations

Several limitations could be identified. Because of the chosen retrospective design of the study, differences in surgical techniques could be found (refixation of subscapularis tendon, fixation of the glenoid bone transplant, PMT). Also, differences in postoperative treatment (early mobilization vs. restrictive protocol) were noted during the analysis. These differences may significantly affect both functional outcome and complication and revision rate. A prospective study design would have been necessary to eliminate this bias. Another limiting factor was the minimum follow-up time of twelve months. Although the average follow-up time was around 2.5 years, some patients were also followed up after one year. Since the risk of prosthesis complications increases with increasing implantation time, some complications may not be detected.

In addition, only a small number of patients were available for follow-up in the clinic. Since fracture sequelae are a rare entity, the number of included patients is high compared to the current literature. Since these are mostly elderly patients with several comorbidities, some patients could not participate. Excluding patients who died unrelated to surgery (17 patients), loss of follow-up was only 24%. Another limiting factor is the formation of subgroups, which results in small group sizes and minimizes statistical power.

Nevertheless, we believe our subclassification is very valuable for the treatment of fracture sequelae of the proximal humerus. To our knowledge, this is the first study to subclassify all fracture sequelae according to Boileau and to give a very detailed overview of the clinical results of our endoprosthetic treatment suggestion in patients of advanced age.

## Conclusion

Due to the diversity of fracture sequelae, a modification of the Boileau classification seems valuable. In this study, a modification of Boileau's classification of sequential fracture sequelae is presented. Due to the diversity and variability of the four types, a subclassification seems to be very useful. For each subcategory, we suggest a specific treatment option. Following the endoprosthesic treatment suggestions, good clinical results can be achieved with a comparatively low revision rate.

## Data Availability

Patient consent does not include publication of the data, so publication of the raw data is not possible. The datasets generated and/or analysed during the current study are not publicly available due not written consent of the patients but are available from the corresponding author on reasonable request.

## Abbreviations

ACS: Age and gender adjusted Constant–Murley Score
AHD: Acromiohumeral distance
AHSA: Anatomic hemi shoulder arthroplasty
ap: Anterior posterior
ASA: Anatomic shoulder arthroplasty
ATSA: Anatomic total shoulder arthroplasty
CS: Constant–Murley score
CT: Computed tomography
EQ-5D-3L: European quality of life 5 dimensions 3 level version
MCID: Minimal clinically important difference
QDASH: Quick disabilities of the arm, shoulder and hand score
ROM: Range of motion
RSA: Reverse shoulder arthroplasty
SSV: Subjective shoulder value
VAS: Visual analog scale
