# Correction to: Surgical treatment of fracture sequelae of the proximal humerus according to a pathology‑based modification of the Boileau classification results in improved clinical outcome after shoulder arthroplasty

**DOI:** 10.1007/s00590-023-03748-y

**Published:** 2023-10-10

**Authors:** Michael Kimmeyer, Jonas Schmalzl, Evelin Schmidt, Annika Graf, Verena Rentschler, Christian Gerhardt, Lars-Johannes Lehmann

**Affiliations:** 1 Department of Traumatology, Hand Surgery and Sports Medicine, ViDia Clinics Karlsruhe, Steinhaeusserstr. 18, 76135 Karlsruhe, Germany; 2 Alps Surgery Institute, Clinique Générale Annecy, 4 Chemin de La Tour la Reine, 74000 Annecy, France; 3 Department of Trauma, Hand, Plastic and Reconstructive Surgery, University Hospital Wuerzburg, Oberduerrbacher Str. 6, 97080 Würzburg, Germany; 4 University of Jena, Bachstr. 18, 07743 Jena, Germany

**Correction to: European Journal of Orthopaedic Surgery & Traumatology** **https://doi.org/10.1007/s00590-023-03721-9**

In the original version of this article, the given names and family names of authors were incorrectly structured.

The given name and family names should have read:

Michael Kimmeyer, Jonas Schmalzl, Evelin Schmidt, Annika Graf, Verena Rentschler, Christian Gerhardt, Lars-Johannes Lehmann

The original article has been corrected.

